# Dynamic control of the dopamine transporter in neurotransmission and homeostasis

**DOI:** 10.1038/s41531-021-00161-2

**Published:** 2021-03-05

**Authors:** Mengfei Bu, Matthew J. Farrer, Habibeh Khoshbouei

**Affiliations:** 1 Department of Neurology and Fixel Institute, University of Florida, Gainesville, FL USA; 2 Department of Neuroscience, University of Florida, Gainesville, FL USA

**Keywords:** Parkinson's disease, Transporters in the nervous system

## Abstract

The dopamine transporter (DAT) transports extracellular dopamine into the intracellular space contributing to the regulation of dopamine neurotransmission. A reduction of DAT density is implicated in Parkinson’s disease (PD) by neuroimaging; dopamine turnover is dopamine turnover is elevated in early symptomatic PD and in presymptomatic individuals with monogenic mutations causal for parkinsonism. As an integral plasma membrane protein, DAT surface expression is dynamically regulated through endocytic trafficking, enabling flexible control of dopamine signaling in time and space, which in turn critically modulates movement, motivation and learning behavior. Yet the cellular machinery and functional implications of DAT trafficking remain enigmatic. In this review we summarize mechanisms governing DAT trafficking under normal physiological conditions and discuss how PD-linked mutations may disturb DAT homeostasis. We highlight the complexity of DAT trafficking and reveal DAT dysregulation as a common theme in genetic models of parkinsonism.

## Introduction

### DAT, a regulator of dopamine neurotransmission and homeostasis

Parkinson’s disease (PD) is the second most common neurodegenerative disease and is best known as a movement disorder. Motor symptoms correlate with the progressive loss of dopaminergic neurons in the *substantia nigra* and Lewy pathology. However, a relative preservation of dopamine synthesis and an increase in turnover is frequently observed in patients early in their symptom onset^1–4^. This supports the hypothesis that dysfunction in presynaptic dopamine neurotransmission may contribute to disease pathogenesis.

Midbrain dopaminergic neurons have a very distinct morphology and mechanism of action. In the rat a single dopaminergic neuron in the *substantia nigra* is estimated to influence ~75,000 striatal neurons via as many as 370,000 axonal boutons, and in humans the organization may be four times as complex^5,6^. Dopamine release can originate from varicosities without termination of the axons, from *en passant* synapses that are often perisynaptic and extrasynaptic. Hence dopamine signaling is not restricted to a single synaptic location but has a large sphere of influence, an architecture that leads to volume transmission^7,8^. Extracellular dopamine critically modulates movement, motivation and learning by acting on its receptor. Different dopamine receptor subtypes display heterogeneity in their regional expression, substrate affinity and G-protein coupling dynamics^9^. Depending on the type of receptor activated, dopamine can have completely opposing effects on target neurons. Volume transmission and dopamine receptor heterogeneity both highlight the need to dynamically regulate extracellular dopamine in time and space - a function diligently executed by the dopamine transporter (DAT, SLC6A3).

DAT is a plasma membrane protein selectively expressed in dopaminergic neurons and critically regulates dopamine neurotransmission^10–12^. As a member of SLC6 gene family of Na^+^/Cl^-^ dependent neurotransmitter transporters, DAT couples the transport of Na^+^ ions down the electrochemical gradient with the reuptake of extracellular dopamine into the intracellular space. Lowering the concentration of extracellular dopamine limits its duration of action and regulates dopamine transmission. Deletion of the DAT gene in mice greatly reduces tissue dopamine content, despite a great increase in the rate of dopamine synthesis^13^, suggesting DAT reuptake is the primary mechanism for maintaining intracellular dopamine storage. Beyond simple reuptake of extracellular dopamine, DAT is capable of generating a depolarizing conductance that regulates the excitability of midbrain dopamine neurons both in vitro and in vivo^14,15^. Under certain conditions, DAT also mediates the release of intracellular dopamine via a reverse transport mechanism known as DAT-mediated dopamine efflux^16^. Recent studies have also identified DAT as a regulator of short-term plasticity in striatal dopamine release^17^.

### DAT structure and modification

DAT contains twelve transmembrane helices that harbor the primary substrate binding site and have large N- and C- terminals extended into the cytoplasm. DAT undergoes N-linked glycosylation on extracellular loop 2, which is important for surface targeting of the transporter. Pathogenic amino acid substitutions have been described near the Na^+^ binding site that interfere with conformational changes during transport, resulting in dystonia-parkinsonism, and which highlight the functional significance of this transmembrane domain^18,19^. Additional rare substitutions have been implicated in attention deficit hyperactivity disorder (ADHD) and autism spectrum disorder (ASD)^20–22^. DAT N- and C- terminals have the most diverse structures among SLC6 family members, and these domains account for differential interaction profiles. Notably, the N-terminus of DAT harbors several secondary modification sites, including residues subject to phosphorylation^23^ and ubiquitination^24^, that are important for the activity and trafficking of the transporter. The C-terminus contains domains for interactions with other proteins including α-synuclein^25^, parkin^26^, flotillin^27^ and protein kinase C (PKC)^28^. Recently, RAS-like GTPase, Rit2 was shown to complex with DAT and regulate PKC-stimulated DAT trafficking^29,30^. Genetic variability in the Rit2 locus has been robustly associated with PD and several other dopaminergic disorders^29,31,32^.

The high-resolution crystal structure of human DAT (hDAT) has not been solved as it is lipid associated and the protein aggregates during purification. Nevertheless, recent studies have resolved a partial structure of Drosophila melanogaster DAT (dDAT) in multiple conformational states. Hence, the functional dynamics of DAT and its interactions with ligands are being elucidated^33^. At resting state, dDAT exists in an open and outward facing conformation. Upon substrate binding, an outward facing occluded conformation forms that is stabilized by salt bridges between key residues (the extracellular gate). Then the occluded complex transitions to become inward facing. Subsequent disruption of a separate set of salt bridges (the intracellular gate) allows substrate release into the intracellular space. Mutations that disrupt the conformational structure often lead to anomalous efflux or inefficient uptake activity^20–22^. However, it should be noted that dDAT intracellular N- and C- termini were removed to facilitate crystallization. In addition, dDAT was crystallized as a monomer^34^, whereas biochemical and microscopy data frequently detect dimers, oligomers and clusters of DAT molecules in DAT expressing model systems^35–41^. Oligomerization could influence DAT trafficking and surface expression. Small molecules, notably AIM100, may directly induce DAT oligomerization and subsequent endocytosis^42^. Readers are referred to the recent review for more detailed discussion on DAT oligomerization^43^.

## Regulation of DAT endocytosis

Plasma membrane dopamine transporters are dynamically regulated by endocytic trafficking. Extensive studies have identified a variety of signaling pathways that lead to DAT internalization. The mechanisms of endocytic carrier formation and post-endocytic sorting of DAT largely depends on the signaling context. In this section, the mechanisms underlying DAT endocytosis are reviewed.

### Clathrin-dependent Endocytosis

Clathrin-mediated endocytosis (CME) was the first mechanism identified for DAT regulation and is best characterized by PKC activation^44,45^, which robustly drives the loss of surface DAT in heterologous systems and striatal tissues^46^. New data suggests PKC activation may disrupt DAT interactions with Rit2, and with cytosolic nonreceptor tyrosine kinase (TNK2, formerly called activated p21CDC42 kinase; ACK1), that normally stabilize DAT in the plasma membrane^30,47^. AIM-100 may also inhibit TNK2 and lower surface DAT^47,48^. The effect of PKC can be arrested by blocking CME. For example, dynamin interacts with clathrin-coated pits and plays an important role in vesicle budding. Expression of dynamin p.K33E, a dominant-negative form of dynamin, or pharmacological inhibition of dynamin activity prevents phorbol 12-myristate 13-acetate (PMA)-induced loss of surface DAT in cell lines and *ex-vivo* striatal slices, respectively^45,49^. Down regulation of clathrin heavy chain by siRNA or acute inhibition of clathrin function by pitstop2 prevents PMA-mediated DAT internalization in DAT expressing cell lines^44,47,50^. However, dynamin is involved in both clathrin-dependent and clathrin-independent pathways including RhoA- and caveolar-mediated endocytosis^51^. Clathrin is also critically involved in a broad range of endocytic pathways including sorting of ubiquitinated proteins for lysosomal degradation^52,53^. Therefore, indirect regulation of clathrin and dynamin on PKC-mediated DAT internalization cannot be excluded. DAT complexes with Eps15, a component of clathrin-coated pits, in heterologous systems and midbrain neuronal cultures^24,50^. However, immunogold-labeling has failed to observed DAT localization in clathrin-coated pits in the axonal terminals and soma of dopaminergic neurons^54–56^. Assessing DAT’s interaction with clathrin and scaffold proteins, such as adaptor protein-2, in endogenously expressing systems may further clarify their role in DAT endocytosis.

### RhoA-mediated Clathrin-independent Endocytosis

RhoA belongs to the Ras GTPase family and defines a clathrin-independent endocytic pathway^57^. RhoA-mediated DAT endocytosis is most studied in the context of substrate exposure including amphetamine (AMPH); AMPH-induced DAT internalization was first described in HEK293 cells^58^, and later confirmed in mid-brain neuronal cultures^59^, *ex-vivo* striatal and mid-brain slices^46,59^. AMPH-mediated DAT endocytosis is dynamin dependent^58^, accompanied by the activation of RhoA and Rac1 in mouse mid-brain slices, and prevented by the inhibition of RhoA through PKA^59^. Similarly, dopamine treatment of rat mid-brain slices also increases RhoA activation and reduces surface DAT expression, and such effects are inhibited by pharmacological activation of the PKA-coupled D1 receptor^60^. AMPH and dopamine-mediated DAT internalization is blocked by cocaine which occupies the substrate binding site of DAT and prevents transport activity^14,16,58,59^. Extracellular AMPH failed to induce internalization of an uptake-impaired mutant form of DAT (Y335A-hDAT); whereas direct intracellular application of AMPH via a whole cell patch pipette restored the trafficking of Y335A-hDAT, and revealed that its endocytic regulation is triggered by intracellular AMPH^61^. Subsequent study has identified the G-protein coupled receptor (GPCR) TAAR1 as the intracellular effector of AMPH-mediated RhoA activation and DAT endocytosis^62^.

An essential downstream effector of RhoA activation is Arp2/3, which directly regulates actin branching and polymerization to provide the necessary force for membrane remodeling in the absence of clathrin^63,64^ and highlights the role of actin dynamics in DAT trafficking. Actin depolymerization agents lead to reduced DAT surface expression in striatal tissue^49^. Piccolo, a component of the presynaptic cytoskeletal matrix, was shown to negatively regulate methamphetamine-induced DAT internalization, possibly through sequestering membrane phosphatidylinositol 4,5-bisphosphate (PIP2)^65^.

### Lipid raft and DAT surface retention

Lipid rafts are cholesterol- and sphingolipid-enriched microdomains on the plasma membrane. DAT is proposed to distribute equally among raft and non-raft domains^49,66^. Although it is generally acknowledged that DAT association with lipid rafts negatively regulates the kinetics of the transporter activity^67,68^ and membrane mobility^27,50,69^, the role of lipid rafts on DAT endocytosis is controversial. While some studies report raft-associated DAT exhibits more resistance to constitutive and PKC mediated endocytosis^66^, others show DAT endocytosis arises equally from raft and non-raft associated populations^49^. DAT forms a stable protein complex with membrane protein flotillin-1, and that interaction appears to be required for DAT targeting to lipid rafts^27,50^. siRNA mediated flotillin-1 knock down may block PMA-mediated DAT endocytosis in heterologous systems and mid-brain dopaminergic neuronal cultures^27^. Paradoxically, in an independent study, the same treatment did not exhibit any effect in PMA-treated HA-DAT expressing cells^50^. Lipid rafts provide a scaffold for plasma membrane protein interactions with the cytoskeleton and promote DAT clustering^40^. It is possible that flotillin facilitates endocytosis by promoting DAT clustering prior to carrier formation, rather than being directly involved in carrier formation, as shown for other cargos^70,71^. However, the conflicting results might be due to the model system used to study raft and non-raft associated DAT trafficking. Future studies might directly examine the trafficking routes of DAT-flotillin complexes in mid-brain dopaminergic neurons expressing DAT endogenously.

## DAT post-endocytic sorting

In general, degradative and recycling pathways are physically separated by distinct endosomal subdomains, and defined by their respective sorting machineries^72^. The endosomal sorting complexes required for transport (ESCRT) family of proteins defines degradative subdomains^73^ and orchestrates the recruitment of ubiquitinated cargos to budding intraluminal vesicles. Conversely, tubular extensions occupy most of the membrane surface and provide subdomains enriched in cargo retrieval complexes, including the retromer and retriever complexes. These generate tubulo-vesicles for plasma membrane recycling or retrograde transport to the Golgi network^74–76^. Extensive efforts have been invested to identify the subcellular localization of internalized DAT, to define its post-endocytic fate. However, the ambiguous colocalization pattern of DAT with known post-endocytic sorting markers, and lack of methodology to reliably track endogenous DAT have led to conflicting results, as reviewed below.

### Degradation

Although the field cannot reach a consensus on the exact post-endocytic fate of DAT, most studies provide evidence that DAT is, at least in part, targeted for lysosomal degradation following endocytosis. Studies using either antibody feeding assays or fluorescent cocaine analogs to visualize epitope-tagged or endogenous DAT in mid-brain neuronal cultures all confirm that internalized DAT partially localizes to late endosomes and lysosomes^77–81^. Electron microscopy studies also show that DAT localizes to the endolysosomal compartment, albeit less in axonal terminals than in soma^54–56^.

N-terminal ubiquitination may be the tag that sorts DAT to its degradative fate. DAT undergoes ubiquitylation on conserved lysine residues in heterologous systems and mid-brain neuronal cultures, which is significantly enhanced following PMA treatment^79,82,83^. Nedd4–2 has been identified to ubiquitinate DAT in a PKC dependent manner^24^. Either down regulation of Nedd4-2 or abolition of DAT N’-terminal lysines by mutagenesis impairs the PKC-mediated endocytosis of DAT^24,83^. However, Nedd4 regulates the trafficking and degradation of many membrane proteins so disruption of DAT trafficking could be a secondary consequence rather than a direct effect.

Interestingly, DAT is proposed to undergo monoubiquitylation and lys63-linked polyubiquitylation^82–84^, both of which serve to target cargos to a degradative fate by recruiting cargo to ESCRT-positive subdomains^85^. Supporting the theory, DAT localization to the ESCRT component ‘hepatocyte growth factor-regulated tyrosine kinase substrate (HRS)-positive’ endosomal compartment was also confirmed in PMA treated YFP-DAT expressing cells^83^. Conversely, modification that reduces DAT ubiquitination also reduces degradation and enhances DAT’s steady state level^86^. Collectively, these studies support the model where PKC-mediated ubiquitination recruits DAT to a degradative fate.

However, attempts to validate PKC-mediated degradation for endogenous DAT have proven challenging as multiple groups report PMA treatment fails to induce DAT internalization in mid-brain neuronal cultures^77,80,81^. Although PKC-induced loss of surface DAT can be robustly reproduced using a biotinylation assay of *ex-vivo* striatal and mid-brain slices, the total level of DAT is not significantly reduced compared to heterologous systems^27,30,49^. Conflicting results may be due to differences in PMA treatment times and/or use of heterologous model systems overexpressing DAT. More research is needed to validate DAT ubiquitination and subcellular localization in its native environments, including the soma and terminal regions of dopamine neurons.

### Recycling

Endocytic recycling pathways for DAT also remain elusive. Early electron microscopy (EM) studies in rodent brains observed DAT immunoreactivity in tubulovesicular structures in the soma, albeit less frequently in axonal varicosities, suggesting DAT may be subject to endocytic recycling^55,56^. Although DAT exhibits partial colocalization with recycling markers, such as syntaxin13, the transferrin receptor and Rab11, in cultured mid-brain neurons and in *ex-vivo* slices, the extent of that colocalization is quite ambiguous compared to *bona fide* recycling cargos such as transferrin and β2-adrenergic receptors^77,80^. Direct comparison between the trafficking of DAT and norepinephrine transporter (NET, a SLC6 family transporter closely related to DAT) shows that NET is more likely to recycle via Rab11 than DAT, due to the non-conserved N’-termini of the two transporters^48^. However, in N2A neuroblastoma cells, expression of dominant-active Rab11 upregulates DAT surface expression and uptake activity^87^. Multiple groups have also demonstrated DAT plasma membrane recycling with antibody feeding or biotinylation based assays in various cell lines and mid-brain neuronal cultures^79,88,89^. In support of its recycling fate, DAT was shown localize to VPS35 positive vesicles in dopaminergic cell lines and dopamine axons in mouse medial forebrain bundle axons^89,90^. Either knocking down or expression of PD-linked mutant Vps35 p.D620N leads to reduced total and surface expression of DAT in heterologous systems and mouse striatal tissues^89,91^, albeit without producing a PD-like motor phenotypes in this animal model^91^. Internalized DAT that undergoes retromer-mediated plasma membrane recycling or retrograde trafficking may potentially account for the comparatively decreased colocalization between DAT and Rab11.

The retromer complex orchestrates sequence-dependent cargo retrieval as opposed to sequence-independent bulk recycling^92,93^. However, the sequence responsible for DAT-retromer interaction remains to be determined. The PDZ domain is a well characterized recycling sequence recognized by retromer and its adaptor protein for plasma membrane recycling^92^. The same mechanism is proposed to regulate retromer-mediated recycling of DAT^89^. Indeed, mice harboring mutations in the putative PDZ domain of DAT exhibit reduced surface DAT expression in the striatum^94^. Furthermore, the DAT PDZ motif was required, at least partially, for exit out of retromer-positive endosomes^89^. However, unlike well-characterized retromer cargos such as beta 2 adrenergic receptor (β2AR) or glucose transporter1 (GLUT1)^95–97^ which have a type I PDZ domain, DAT has a type II PDZ domain. This is unlikely to be recognized by SNX27, the adaptor protein best characterized in retromer-mediated plasma membrane recycling^98,99^. Retromer binding is sometimes facilitated by palmitoylation of cysteine residues in the cytosolic tails of cargo proteins^100^, and notably, DAT undergoes C’-terminal cysteine palmitoylation^101^. Pharmacological inhibition or mutagenesis of those cysteine residues enhances PMA-induced downregulation of DAT in heterologous cells and rat striatal synaptosomes, suggesting palmitoylation may function to resist PKC-mediated DAT degradation^101^. The functional impact of palmitoylation on dopamine reuptake kinetics of surface DAT is well described^102^, but whether palmitoylation affects DAT endocytic sorting and trafficking is unknown.

## DAT axonal targeting

Another puzzle in DAT trafficking is the mechanism of DAT axonal targeting. Midbrain dopaminergic neurons project long and extensively arborized terminals^5^. In rat a single dopaminergic neuron in the *substantia nigra* is estimated to influence ~75,000 striatal neurons^5^, via as many as 370,000 terminal axonal boutons^6^. DAT is found throughout the plasma membrane of the axons and varicosities of dopaminergic neurons^54–56^. It seems intuitive that with such a complex morphology, dopaminergic neurons might employ local DAT synthesis rather than long-range axonal trafficking. Surprisingly, the *in-situ* hybridization signal for DAT mRNA is primarily observed in the mid-brain and almost invisible in the striatum^103,104^, although that may reflect the probes used and the sensitivity of traditional *in-situ* methods. It remains possible that DAT mRNA may be found outside of the somatodendritic compartment. At the protein level, most DAT localizes to smooth endoplasmic reticulum in the soma and not in axonal terminals^54–56^. In mid-brain neuronal cultures from tagged HA-DAT knock-in mice, HA-DAT vesicles display rapid bi-directional movement along axons suggesting that DAT is synthesized in somal endoplasmic reticulum (ER) and subject to axonal trafficking. A recent study examined long-range DAT trafficking in medial forebrain bundle axonal tracts to reveal that most DAT is membrane localized with a more limited distribution in retromer positive vesicles^90^. The result raised the possibility that DAT might be trafficked by both membrane diffusion and vesicular transport^90^. Future studies examining DAT localization to microtubule markers and/or microtubule motor proteins such as kinesin would further clarify the mechanism of long-range DAT trafficking.

ER export relies heavily on coated-protein complex (COPII) coat-derived vesicles with SEC24 family proteins orchestrating cargo recognition^105^. Supporting the role of SEC24-mediated ER export in DAT axonal targeting, siRNA knockdown against SEC24D in heterologous cell lines slows down dopamine reuptake^106^. In C.*elegans*, deletion or mutagenesis of a conserved sequence ^591^PYRKR^595^ in the DAT-1 C′-terminus leads to somatic retention^107^. The PYRKR sequence contains a 3 R motif that mediates the interaction between SEC24 and α2β-adrenergic receptor^107,108^, suggesting the failure of DAT-1 synaptic localization might be due to reduced interaction with SEC24-1. In HEK293 cells, mutation of the corresponding sequence in DAT does not affect its interaction with SEC24D, nor significantly reduces DAT surface expression, but does disrupt the ability of SEC24D to enhance DAT surface expression^107^. However, HEK293 cells are not going to be an informative model for DAT axonal trafficking as they lack the bipolar morphology of dopaminergic neurons.

**Fig. 1:**
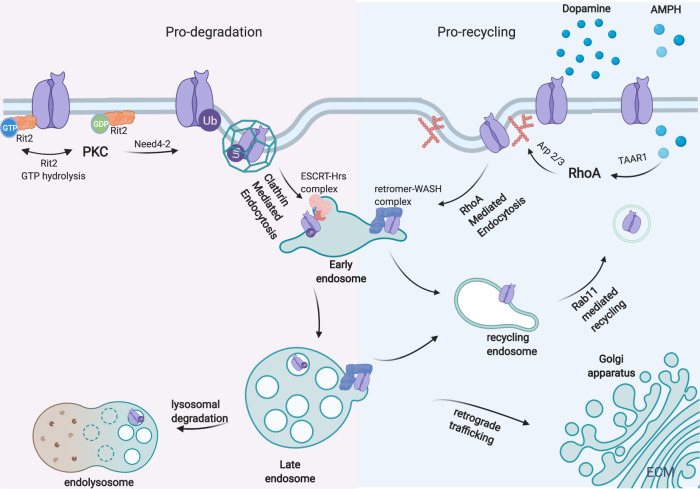
Dopamine transporter surface expression is regulated through endocytic trafficking. RIT2-DAT association acts as DAT endocytic brake. PKC activation promotes the dissociation of RIT2 from DAT, enhances DAT ubiquitination by Nedd2-4, and targets DAT to clathrin-mediated endocytosis. Ubiquitinated DAT is recruited to ESCRT-Hrs complex which sorts DAT for lysosomal degradation. Conversely, DAT substrate activates intracellular effector TAAR1 and RhoA GTPase, which orchestrates actin polymerization through Arp2/3 near the plasma membrane and mediates clathrin-independent endocytosis. Substrate induced DAT internalization preferentially targets DAT to Rab11 mediated plasma membrane recycling, possibly through retromer-WASH (the endosomal cargo retrieval complex). This figure is created with BioRender.com.

In conclusion, multiple molecular mechanisms are implicated in DAT trafficking and the specific trafficking route is highly dependent on signaling. Key mechanisms are summarized in Fig. 1. DAT trafficking studies have suffered from a lack of reagents to reliably track DAT in its native environment, and work conducted in heterologous systems had often produced conflicting results. More recent super-resolution microscopy has revealed the dynamic localization of neuronal transmembrane proteins, including DAT^40,109,110^, and may prove useful to dissect mechanisms governing endogenous DAT trafficking in *bona fide* dopaminergic terminals.

## The dopamine transporter, monogenic parkinsonism, and models

Given the complex morphology of dopaminergic neurons and the onerous burden of long-term axonal trafficking, it is tempting to speculate that endocytic dysregulation of the dopaminergic transporter may lead to the selective vulnerability of dopaminergic neurons in PD. In this section, monogenic parkinsonism, and models based on those gene linkages are reviewed, albeit focused on their DAT biology. Most mice that model human gene mutations linked to PD do not exhibit overt motor symptoms but many have subtle alterations to their dopaminergic system, as illustrated in Table 1.

**Table 1:** Mouse models of genes implicated in Parkinson’s disease and their dopamine neurotransmission phenotypes.

Gene	Model	Motor symptom	Dopamine neurotransmission phenotype	Proposed role in DAT regulation
SNCA	mThy-1 human WT α-synuclein overexpression^112^	L-Dopa responsive sensorimotor deficits	Increased extracellular dopamine; increased dopamine turnover rate; lower striatal TH level	Membrane microdomain targeting^121^, and surface insertion^25,122^.
	(AAV6)– human WT α-synuclein^116^	Age-dependent motor deficits	Reduced striatal dopamine release and dopamine reuptake; reduced dopamine tissue levels;	
	CaM-tTA human WT α-synuclein overexpression^115^	Age-dependent motor deficit	Significant reduction of dopamine level in the olfactory bulb; Trend towards reduced dopamine in striatum; loss of dopamine neurons in SN.pc	
	MPrion human A53T α-synuclein overexpression^113,114^	Motor hyperactivity	Reduced DAT expression and dopamine reuptake; Increased dopamine content in striatal tissue; no evident loss of dopamine neuron in SN.pc	
LRRK2	BAC human LRRK2 overexpression^176^	Motor hyperactivity	Elevated striatal dopamine release; comparable dopamine uptake and tissue dopamine content; no loss of dopamine neurons in SN.pc	Long term axonal trafficking^132^, endolysosomal trafficking^149^
	BAC human LRRK2 R1441G^177^	L-DOPA responsive motor deficits	Reduced striatal dopamine release; no evident loss of dopamine neuron in SN.pc	
	Human LRRK2 G2019S transgene^178^	Age-dependent motor deficits	Normal striatal dopamine level and turnover rate; Increased dopamine turnover rate in olfactory bulb; Age-dependent loss of dopamine neurons in SN.pc	
	Mouse Lrrk2 R1441C knock-in^179^	Decreased locomotor responses to quinpirole; reduced locomotor response to AMPH	Normal striatal dopamine level; reduced catecholamine release; no loss of dopamine neurons in SN.pc	
	Mouse Lrrk2 G2019S knockin^127,128^	Early hyperactivity that declines with age^128^	Reduced striatal dopamine release; trend of reduced DAT expression; age-dependent DAT dysfunction; increased dopamine turnover rate; no loss of dopamine neurons in SN.pc	
VPS35	Mouse Vps35 D620N knockin^91^	No evident motor symptoms	Altered striatal dopamine release; reduced dopamine reuptake; reduced striatal DAT expression; no evident loss of dopamine neurons in SN.pc	DAT endocytic sorting and recycling^89,91^
PARKIN	Mouse Parkin knockout^158,159^	Reduced spontaneous motor activity	Increased dopamine turnover; Increased extracellular dopamine; Reduced striatal dopamine release; reduced dopamine reuptake; no loss of dopamine neurons in SN.pc	Protein quality control^26^; endolysosomal trafficking^155,156^
RIT2	Mouse *Pitx3*^*IRES2-tTA*^ *RIT2 knockout*^30,166^	Increased acute cocaine sensitivity in males, but decreased in females	In females: reduced surface DAT expression in ventral striatum; In males: reduced total DAT levels; increased [^3^H] dopamine reuptake in striatal slices	DAT surface retention^29^

The literature cited is not exhaustive. However, reduced DAT-mediated dopamine reuptake is frequently observed in mouse models without dopaminergic degeneration.

### SNCA

α-synuclein (SNCA) is a presynaptic protein and a major component of Lewy pathology. Studies of familial forms of Parkinson’s disease have identified *SNCA* missense mutations and genomic multiplications^111^. Mice models overexpressing mutant or wild type human α-synuclein mostly exhibit reduced DAT expression and dopamine reuptake^112–116^ with some exceptions^117^. α-synuclein may form a stable complex with DAT in heterologous systems, rodent and human striatal tissues^25,118,119^. Studies in dopaminergic neuronal cultures suggest DAT/α-synuclein association negatively regulates DAT reuptake activity^120–122^ possibly by redistributing surface DAT to cholesterol-rich microdomains and altering ionic coupling of DAT mediated current^120,121^. DAT surface expression, measured by biotinylation, is also reduced with co-expression of wild type α-synuclein^123,124^. When compared to wild type α-synuclein, the PD-linked missense mutation *SNCA* p.A30P enhances DAT α-synuclein interaction and further reduces DAT surface expression and function; in contrast, p.A53T does not form a strong protein-protein complex with DAT, nor modulates DAT function or surface expression. Although *SNCA* p.A30P and p.A53T have disparate effects on DAT-binding and trafficking, they are N’-terminal located on the hydrophobic face of α-synuclein’s amphipathic helix, highlighting the importance of maintaining that protein-lipid interaction.

### LRRK2

Leucine-rich repeat kinase 2 (LRRK2), encodes a large multidomain protein containing kinase and GTPase enzymatic activities. LRRK2 genetic variability is found throughout the gene but pathogenic mutations cluster in the Roc-COR and kinase domains, and account for this common form of familial parkinsonism^111^. All mutations lead to upregulation of LRRK2 kinase activity. Investigations of in vivo dopaminergic function with PET imaging consistently reveal increased dopamine turnover rates in asymptomatic LRRK2 heterozygotes^1,2,125^. Interestingly, those asymptomatic individuals showed reduced DAT levels, measured by the commonly used PET tracer 11C-d-threo-methylphenidate (^11^C-MP)^125^, suggesting early dysfunction of DAT in the absence of significant loss of dopamine synthesis and storage. In rodent models, temporal overexpression of human LRRK2 p.G2019S in dopaminergic neurons impairs DAT mediated dopamine reuptake^126^. Constitutive Lrrk2 p.G2019S knock-in mice also exhibit impairment in striatal dopamine reuptake at various ages^127,128^. Although there are limited number of studies that directly examine the role of LRRK2 in DAT regulation, growing evidence has established LRRK2 as an important regulator of intracellular membrane trafficking^129^, at a nexus between actin-dependent and tubulin-dependent cargo transport^130–132^. Mutant LRRK2 leads to dysregulated endocytic sorting^133^ through phosphorylation of Rab proteins^134,135^ whose coordinated action critically regulates vesicular transport and endosome maturation^136,137^. LRRK2 binding to microtubules is also associated with tau phosphorylation^138,139^, microtubule acetylation^132,140^ and impaired axonal transport^132^. Hence, DAT endosomal recycling and axonal trafficking may be dysregulated by chronic LRRK2 hyperactivity. Exploring the increased (or compensatory) dopamine turnover observed in asymptomatic LRRK2 heterozygous individuals may provide mechanistic insight into the physiological and pathological role of LRRK2 in DAT regulation.

### VPS35

VPS35 is a component of the retromer complex required for recycling cargo protein in the early endosome to the trans-Golgi network or plasma membrane^76^. A missense mutation, VPS35 p.D620N has been linked to a familial form of parkinsonism that is clinically indistinguishable from idiopathic late-onset PD^141,142^. VPS35 colocalizes with DAT and mediates DAT plasma membrane recycling both in vitro and in vivo^89^. Mice harboring Vps35 p.D620N exhibit reduced striatal DAT expression and altered striatal dopamine neurotransmission in the absence of motor deficits or neurodegeneration^91^. Although the exact pathological mechanism of VPS35 p.D620N remains elusive, multiple studies show the mutation disrupts the interaction between VPS35 and the WASH complex^143,144^ whose primary function is to coordinate actin branching and stabilize endosomal subdomains for VPS35-mediated cargo sorting of recycling proteins^145^. Failure to adequately recruit or retain WASH complex may lead to the mis-sorting of DAT, as illustrated by other retromer cargos^143,145,146^. These data are consistent with the hypothesis that expression of VPS35 p.D620N can impair DAT recruitment and recycling.

Increasing evidence suggests that VPS35 and LRRK2 functionally interact and converge on endocytic trafficking. VPS35 and LRRK2 directly interact, decorate endosomes, and may work in concert in synaptic vesicle recycling^147^. VPS35 p.D620N significantly elevates LRRK2-mediated phosphorylation of Rab proteins in mouse brain and human neutrophils^148^. Endolysosomal dysfunction induced by mutant LRRK2 can be rescued by overexpression of VPS35^149^, although toxic to synapses in primary culture^96^. Given VPS35 and LRRK2 mouse models exhibit similar deficits in DAT mediated reuptake, it is possible that they also cooperate in the regulation of DAT trafficking. Interestingly, acute treatment with the LRRK2 kinase inhibitor, MLi2, in Vps35 p.D620N knock-in mice rescues the reduction in striatal DAT levels and the reduction in dopamine reuptake^91^ (Farrer lab, unpublished data). However, it is currently unknown which step in DAT trafficking (e.g., endocytosis, endocytic sorting, axonal targeting) is involved and whether VPS35 and LRRK2 work in concert or in competition.

### Parkin

Parkin is a protein-ubiquitin E3 ligase which ubiquitinates and targets substrate to proteasome degradation^150^. Mutations in Parkin lead to loss of function of its ubiquitin ligase activity and are linked to autosomal recessively inherited, early-onset Parkinson’s disease with a central role in mitophagy^151^. However, patients with mutations in Parkin often exhibit a marked reduction of DAT expression or uptake activity^152,153^. DAT ubiquitination was previously shown to be increased in Parkin transfected human dopaminergic SH-SY5Y cells^26^. WT Parkin, but not PD-linked mutants, significantly increase DAT surface expression and dopamine reuptake, and possibly enhance degradation of misfolded DAT^26^. An association between Parkin and DAT has also been shown to disrupt the interaction between DAT and α-synculein^154^; however, further validation using a more sensitive approach, such as Förster resonance energy transfer (FRET) or yeast 2-hybrid methods, is warranted. Parkin is reported to ubiquitinate Rab7, Eps15, and VPS35 and so contributes to the regulation of the endo-lysosomal pathway^155–157^. Parkin knock-out mice exhibit reduced striatal levels of DAT and dopamine uptake^158,159^. In patient-derived IPSCs, loss of Parkin function also leads to reduced [^3^H]CFT (2-β-carbomethoxy-3-β-(4-fluorophenyl)tropane, a cocaine analog) binding to DAT and increased Ca^2+^-independent dopamine release, potentially via a reverse transport mechanism^160^. Several studies support the hypothesis in which loss of parkin function reduces DAT activity either directly, through impaired protein quality control, or indirectly through dysregulation of DAT endocytic trafficking.

### RIT2

*RIT2* was identified as a PD risk gene in multiple GWAS studies and in different ethnic groups^31,32,161–164^, and Rit2 expression is significantly reduced in the *substantia nigra* of PD patients^165^. Rit2 encodes a small neuronal GTPase that associates with DAT and may act as DAT endocytic brake^30^. Studies in heterologous systems reveal RIT2 associates with the C-terminus of DAT, but not SERT, and that RIT2 GTPase activity is critical for PKC-mediated DAT internalization^29^. Although Rit2 was shown to co-immunoprecipitate with DAT in mouse striatum^29^ this result appears to be an artifact as the antibody used was unspecific^30^. Nevertheless, the effects of Rit2 in *bona fide* dopaminergic terminals are confirmed in mouse striatal slices^30,46^. In *Pitx3*^*IRES2-tTA*^ shRit2 mice, where Rit2 is selectively knockdown in dopaminergic neurons, PKC-stimulated DAT internalization in acute striatal slices is significantly reduced^30^. Interestingly, *Pitx3*^*IRES2-tTA*^ shRit2 mice exhibit sex-dimorphism in dopamine related phenotypes^166^. Conditional Rit2 knockout leads to a blunted response to acute cocaine treatment in female mice, but an increased response in males^166^. Accordingly, [^3^H] dopamine uptake in male striatal slices is enhanced without significant change in surface DAT expression^166^, whereas in females surface DAT is reduced in the ventral striatum^30^. Considering the important role of sex in DAT function and PD risk^167–172^, RIT2 poses a promising target to study the effect of sexual dimorphism in DAT regulation and in the etiology of PD.

## Concluding remarks

**Fig. 2:**
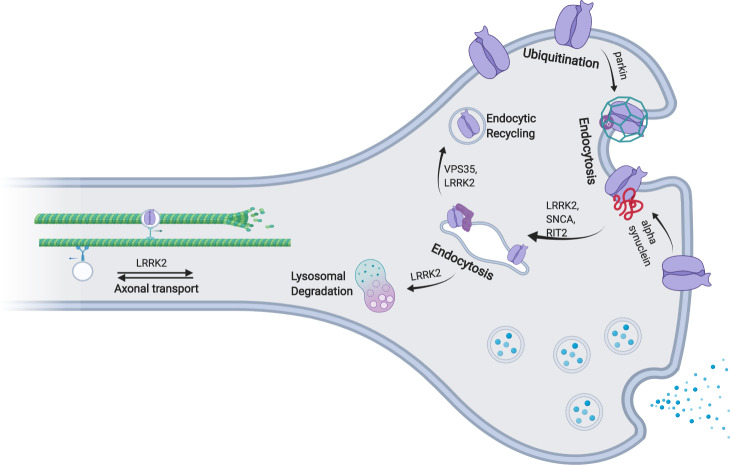
A model for the physiological and pathological roles of PD-lined genes in DAT regulation. Dopamine transporter trafficking is regulated by various mechanisms, many of which overlap with key molecular mechanisms compromised by PD-linked mutations. α-synuclein and Rit2 interact with DAT and regulate both surface expression and dopamine uptake kinetics. LRRK2 may regulate DAT trafficking either through axonal transport and/or endocytic trafficking. Parkin is proposed to ubiquitinate improperly folded DAT and increase its turnover rate. VPS35 is responsible for recruiting internalized DAT to a recycling fate. This figure is created with BioRender.com.

A variety of signaling pathways have been identified that dynamically regulate dopamine reuptake through DAT plasma membrane trafficking, and many overlap with key molecular mechanisms compromised by PD-linked mutations as illustrated in Fig. 2. Down regulation of DAT-mediated dopamine reuptake and increase in striatal dopamine release are often observed in genetic models of parkinsonism. Why would the neurons employ so many complicated signaling pathways to regulate DAT trafficking? DAT critically maintains dopamine homeostasis to regulate dopamine signaling that is absolutely required for movement initiation and survival. Cytosolic dopamine is also easily oxidized, may lead to the production of free radicals, and drives neuromelanin biosynthesis^173^. Direct administration of dopamine and overexpression of dopamine transporter both lead to neuronal loss^174,175^. Conversely, dopamine reuptake by DAT is the primary mechanism for the maintenance of dopamine storage, and loss of DAT leads to dopamine depletion^13^. The depolarizing currents generated by dopamine reuptake also directly regulate the excitability of presynaptic boutons^15^. Thus, DAT surface expression must be controlled to allow dopaminergic neurons to optimally function. The complexities of DAT regulation may enable multiple compensatory mechanisms to maintain dopamine signaling despite early dysfunction. Given the role of DAT in the regulation of dopamine release and the long-hypothesized connection between altered dopamine neurotransmission and PD, studies of DAT regulation in physiological and pathological conditions remain a promising avenue to identify early biomarkers and therapeutic targets for PD.
